# Information–Thermodynamic Method for the Study of Proliferation of Organized Cellular Structure

**DOI:** 10.3390/cells12050731

**Published:** 2023-02-24

**Authors:** Vyacheslav T. Volov, Larisa T. Volova, Alexander V. Kolsanov

**Affiliations:** Ministry of Health of Russia, Research Institute of Biotechnology “Biotech”, Samara State Medical University, 443099 Samara, Russia

**Keywords:** conditional entropy of the cell proliferation, fractal dimension, organized cell structure, juvenile fibroblasts, thermostat, pulsed electromagnetic impact

## Abstract

The aim of the article was to develop an innovative method for the study of cell proliferation based on the information–thermodynamic approach, including the mathematical ratio—the entropy of cell proliferation and an algorithm for the calculation of fractal dimension of the cellular structure. Approbation of this method with pulsed electromagnetic impact on culture in vitro was implemented. It is shown on the basis of experimental data that the organized cellular structure of juvenile human fibroblasts is a fractal. The method makes it possible to determine the stability of the effect on cell proliferation. The prospects for the application of the developed method are discussed.

## 1. Introduction

The current stage of development of the biomedical sciences’ field in the world is characterized by an intensive expansion of the use of natural and mathematical research methods, the use of new approaches, technologies for the diagnosis and treatment of socially significant diseases, on the one hand, and the active introduction of digital technologies, on the other.

At the same time, at first glance, the use of non-invasive technologies is put forward, which can significantly improve the results of treatment and improve the quality of patients’ life. In particular, in regenerative medicine, the role of non-invasive methods associated with the use of magnetic fields in medical practice is increasing. At present, constant, variable, and pulsed magnetic fields (CMF, VMF, and PMF) with low values of magnetic induction (up to 100 mT) are widely used [1,2,3,4,5,6,7,8].

According to most authors [5,6,7,8], the biological and therapeutic effect of magnetic fields is based on changes in the state of cell membranes, enzymatic and receptor molecules, and an increase in permeability of the cell plasmolemma. As we stated above, the use of the universal tools of thermodynamics in its informational interpretation is of particular importance.

It should be noted that the use of thermodynamic methods for studying the development of biological organisms and, in general, reliance on the energy paradigm in this area, have a rather long history.

First of all, this fact is connected with the problem of studying the organism’s own (individual) time, which begins with the classical works of M. Rubner [9] and his followers [10,11,12,13,14]. Besides this, works are presented from studies of the late XX-early XXI centuries [15,16,17,18,19,20,21,22,23], where, in particular, attempts were made to solve this problem by using the thermodynamic tools. The realization that a living organism exists in its own time, the flow rate of which depends on various processes occurring in the organism, was discovered a long time ago. One of the first attempts to formalize the notion of an organism’s own time was made by G. Bakman [11].

The definition of one’s own (physiological) time, based on the process of metabolism, was proposed by J. Reiss [24]. As the basis of his own (physiological) time, he took the concept of “absolute metabolic scope” introduced by M. Boddington [25]—a constant equal to the product of the maximum duration of life and the rate of basal metabolism divided by a unit of mass. This constant represents the minimum amount of energy consumed by a unit of organism’s mass during its lifetime and does not depend on the absolute value of the organism’s mass.

Based on the hypothesis proposed by Rubner about an approximately equal amount of energy used by a unit of mass of an adult animal for the period of life, E. Bauer [10] extended this equality of energy consumption per unit of mass of an animal for the entire period of life and for animals of other levels of organization. Bauer [10] called this quantitative measure the Rubner’s constant, which was defined as the product of the basic exchange per day for the duration of life in days divided by the organism’s weight. Nevertheless, later Bauer confirmed the illegality of the extension of the proposed concept and suggested that the Rubner’s constant is a criterion for an animal belonging to a certain level of evolutionary development and is determined by approximately the same free energy of germ cells inside it. With the increasing complexity of the organization of animals in the process of evolution, this constant increases.

An analysis of the scientific literature on this issue leads to the conclusion that, in most cases, this concept is not given the meaning that it originally had. In the biological literature of the late XX-early XXI century, the Rubner’s constant is often called the total specific metabolism for the entire period of an animal’s life [15,16,17,18,19,20,21,22,23]. A new impact for the application of the universal apparatus of thermodynamics in the study of biological organisms came with the creation of synergetics in the second half of the XX century, and more specifically its component—the non-equilibrium thermodynamics of the structure I. Prigogine [26].

The analytical toolkit of thermodynamics of the structure [26], which was created as the part of synergetics, was primarily used to describe biological systems, i.e., open thermodynamic systems, which are placed far from equilibrium. Concepts, such as entropy, make it possible to investigate the stability of positive and pathological processes occurring in vivo and in vitro.

In the works of A.I. Zotin [15,16,17,18,19,20,21], A.A. Zotin [18], based on the analytical apparatus of I. Prigogine’s thermodynamics, a cycle of studies of the organisms’ development devoted to the problems of aging was carried out. In the studies of A.F. Alimov and his collaborators [22,23], in particular, a significant discrepancy in the values of the Rubner’s constant for different types of organisms was shown. The development of this approach under using of a synergistic apparatus, which allows us to describe the behavior of complex self-organizing systems, which include biological organisms and organized cellular structures, was the work of one of the authors of this text (V.T. Volov) [27].

The developed mathematical measure satisfied the rules of the constructed mathematical entropy [28] based on data on the metabolism and growth of various organisms [15,16,17,18,19,20,21,22,23].

The entropy invariant [27] made it possible to generalize the development of biological organisms not only by their metabolism, but also by the organism’s growth. In this case, the discrepancy between the values of the entropy invariant for organisms does not exceed 2–3%, while, as noted above, the change in the Rubner’s constant will be an order of magnitude higher.

In connection with this fact, the article proposes an innovative method for the analysis of cell proliferation based on the information–thermodynamic approach (the entropy and structural criteria), as well as testing this method based on experimental data on the cell growth under electromagnetic exposure.

## 2. Materials and Methods

### 2.1. Mathematical Methods for the Studying of Cellular Environment

The apparatus of mathematical statistics, the formalism of the theory of mathematical entropy [28], the criteria of fractal geometry [29,30,31], and non-equilibrium thermodynamics of the structure of I. Prigogine [26] (criteria for the stability of a self-organizing system—the theorem on the minimum of entropy production) were used as tools for the studying of cell proliferation. In addition, morphological criteria for the cell proliferation were used [32,33].

### 2.2. Experimental Methods for the Studying of Cell Proliferation

Cellular research methods. The study used data from [33,34,35,36,37], where dermal fibroblasts were isolated by migration from the skin fragments obtained during circulation surgery in boys under the age of 10 years according to the method of Greenberg et al. [32]. The studies using cell cultures were carried out at the Research Institute of Biotechnology “Biotech” of the Samara State Medical University.

Primary material [33,34,36,37] was obtained from donors after signing a voluntary informed consent by legal representatives and approval of the study by the Bioethics Committee of the Samara State Medical University of the Ministry of Health of Russia (protocol No. 184 dated 3 May 2017). The donors were somatically healthy, tested for HIV, syphilis, hepatitis B, and C; test results are negative.

The cell cultivation in the above work was carried out in nutrient medium 199 (Biolot, Russia) containing 10% FTS (Biolot, Russia) and 40 µg/mL of gentamicin (Dalkhimpharm, RF). Fibroblasts were cultured in vitro up to the 20th passage, frozen at a concentration of 1.5 × 10^6^, and stored in cryoprobes (Nunc, Waltham, MA, USA) in Dewar flasks with liquid nitrogen. Thawed cells were seeded first on a T25 flask, and the vial was transferred to a 96-well tablet. Fibroblasts of 20–24 passages were used for experiments. The cell phenotyping was performed using the flow fluorescent cytometry.

Morphological analysis. The cell morphology was assessed by the phase contrast microscopy using an inverted microscope (Olympus CKX41 (Japan)).

The proliferation index (IP) was determined by the following formula:

(1) $$IP=\frac{N_{t}}{N_{0}},$$

where $N_{0}$ is the initial number of cells in the monolayer; and $N_{t}$ is the number of cells on the plastic after 24 h. The doubling time (TD) was determined by the following formula:

(2a) $$TD=t\frac{\mathrm{lg}2}{\mathrm{lg}(N_{t}/N_{0})},$$

where t is the incubation time; $N_{0}$—initial dose of cells on plastic; and $N_{t}$ is the number of cells that have grown during time t. The number of doublings is determined by the formula:

(2b) $$KD=(\mathrm{lg}N_{t}-\mathrm{lg}N_{1})/\mathrm{lg}2.$$

### 2.3. Entropy-Structural Method for the Dynamic Assessing of Cell Proliferation

According to the popular expression of one of the founders of synergetics, G. Haken, the bases of all processes are the differences (gradients) of entropy. Therefore, the development of an entropy toolkit for the analyzing of organized structure proliferation is extremely important and relevant.

In accordance with the theory of mathematical entropy [21], the construction of a deterministic measure on the set—the entropy of cell growth—must satisfy the following conditions:

(3) $$\tilde{H}=\sum_{1}^{N}{\tilde{H}}_{i}—\mathrm{additivity};$$

(4) $$\infty >\tilde{H}>0—\mathrm{limitation}\;\mathrm{and}\;\mathrm{positivity};$$

(5) $$H=\tilde{H}/{\tilde{H}}_{\max}\subset (0,1)—\mathrm{an}\;\mathrm{entropy}\;\mathrm{normalization}.$$

Let us introduce the deterministic entropy of cell growth in organized cellular structures in the following form:

(6) $$H\left(t\right)={\left(1-\frac{1}{\ln\frac{\rho_{\min}}{\rho_{\max}}}\ln\frac{\rho \left(t\right)}{\rho_{\max}}\right)}^{\mu },$$

where $\rho_{\max},\;\rho_{{}_{\min}},\;\rho (t)$ are the maximum, minimum, and current values of the density of human dermal cells (fibroblasts, cancer cells, etc.), respectively.

The coefficient in Formula (4) is determined for each cell type under control growth conditions using the entropy invariant for the calibration [22]:

(7) $$\frac{1}{\Delta T}\int_{0}^{\Delta T}H\left(t\right)dt=H_{0}=0.618,$$

where ΔT is the period of cell proliferation time. The entropy of waste products of a cell culture (waste products) is determined by analogy with (6) using the following formula:

(8) $$H^{*}(t)=\left(1-1/(\ln(m^{*}{}_{\min}/m^{*}{}_{\max}\right))\cdot \ln(m^{*}(t)/m^{*}{}_{\max}){)}^{\mu },$$

where $m^{*}{}_{\min},m^{*}{{}_{\max}}^{}$ is the maximum and minimum value of the mass of waste, respectively, and the value is determined during calibration for the control series in the same way as it is realized for the cell growth (6).

The second important criterion for the studying of evolutionary dynamics of cell cultures is the criterion of fractal dimension—Hausdorff’s criterion.

In the natural sciences, the term “fractal structure” refers to the fractional power dependence between physical quantities in the process or phenomenon under study:

(9) $$X=Y^{D},\;\mathrm{where}\;D\geq 1.$$

The value of D can be interpreted as a formal analogue of fractal dimension. The method for measuring of fractal dimension based on Hausdorff’s criterion [10,11,12] is as follows. On the basis of cell culture images, the ratios of areas (colored cell and unpainted intercellular space) are determined, an approximate measure of which can be obtained using the following formula:

(10a) $$S_{\exp}=S^{D/a},$$

where $S_{\exp}$ is the measured area of painted culture surface, S is the total surface of the image, D is an analogue of the fractal dimension, and a=2 is the dimension of a flat space. After calculating the colored area of culture, $S_{\exp}$, an analogue of the fractal dimension, Hausdorff’s dimension (D) of organized cell structure in vitro, is determined.

The ratio of values D on different scales (with decreasing) is calculated. If, at the same time, the value of

(10b) $$D=a\cdot (\log S_{\exp})/\log S$$

remains unchanged (within the limits of experimental accuracy), then it can be interpreted as an analogue of fractal dimension—Hausdorff’s dimension of an organized cell structure in vitro.

It should be emphasized that this criterion makes it possible to determine the state and dynamics of changes in the organized cellular structure, including cell proliferation, cellular environment, and cell waste products.

#### Energy Balance of the Cellular Structure

As is known, for the use of physical medicinal effects in vivo, it is necessary to conduct appropriate studies in vitro.

For this purpose, studies of the organized cellular structure are carried out in the laboratory. The cellular structure consists of a nutrient medium in which proliferating cells are placed under thermostat conditions. In a 96-well plate, an organized cellular structure with a height of about $h\approx 4\times 10^{-6}$ m was placed in each hole with a diameter of $d=3\times 10^{-2}$ m.

The heat exchange in cellular structure with the environment is carried out in a thermostat at a temperature T=310 K. The organized cellular structure is an open thermodynamic system, the mass of which remains constant during the cell proliferation.

Analogous to the equations of the energy balance of a cell culture for a control culture and for the cell culture under electromagnetic influence are the following in difference form:

(11a) $$\Delta E^{heat}+\Delta E^{feed}=a_{d}{\tilde{D}}_{control}{\left(1/\rho_{\min}-1/\rho_{\max}\right)}_{control}+cM_{st}\Delta T$$

(11b) $$\delta E^{external}+\delta E^{heat}+\delta E^{feed}=a^{*}{}_{d}{\tilde{D}}_{ext}(1/\rho_{\min}-1/\rho_{\max})+cM_{st}\Delta T,$$

where Equation (11a) refers to the control culture, (11b) is the balance equation for the culture with exposure, $\Delta E^{heat}$, $\Delta E^{external}$ are portions of the thermal energy of the environment and pulsed electromagnetic energy obtained over a fixed period of cell proliferation, respectively, $\Delta E^{feed}$ is the energy of nutrient medium obtained over a fixed period of cell proliferation, ${\tilde{D}}_{control}$, ${\tilde{D}}_{ext}$ are the average values of the Hausdorff’s dimension of culture in the control culture and when exposed, respectively, for a fixed period of time of the proliferation cycle Δt, $a_{d},\;a_{d}^{*}$ are the proportionality coefficients for the natural cell proliferation and when exposed, respectively, having the dimension of energy, cell proliferation, for a fixed period of time of the proliferation cycle, $\rho_{\min},\;\rho_{\max}$ are the values of cell distribution densities at the initial and final moments of the experience, $1/\rho_{\min},\;1/\rho_{\max}$ are the corresponding specific areas of their distribution, $M_{st}$ is the mass of the organized cell culture, ΔT is the temperature difference between the external environment and the organized cell culture, and c is the coefficient of heat capacity of the cell culture. Consideration of the process of fibroblast proliferation in two-dimensional space is due to the fact that the thickness of cell structure is several orders of magnitude smaller than other dimensions of the structure (h/d<<1), where h is the height of structure, and d is its characteristic size. The first term on the right side of Equations (11a) and (11b) represents an analogue of the expansion operation in classical thermodynamics (pdv, where p is pressure, v is volume). The second term on the right side of Equations (11a) and (11b) represents the internal energy of culture. The mass of cell culture consists of the mass of cells $M_{cell}$ and the mass of intercellular medium $M_{medium}$:

(12) $$M_{st}=M_{cell}+M_{medium},$$

The mass of proliferating cells changes over time according to the law:

(13) $$M_{cell}(t)=M_{cell0}\cdot f(t),$$

where f(t) is the empirical function of cell proliferation in the control culture series, $M_{cell}(t)$ and $M_{cell\;0}$ are the current and initial value of cell mass, respectively, while $M_{cell0}=m_{cell}\cdot N_{cell0}$. The mass of the intercellular medium depends on time and is determined by the equation:

(14) $$M_{medium}=M_{feed}(t)+M_{wasteproducts}(t),$$

where $M_{feed}(t)\;$ and $M_{wasteproducts}(t)$ are empirical dependences of changes in the mass of the nutrient medium and the products of cell activity.

The proliferative mass of cells under exposure can be determined based on the analysis of experimental data [16] by the equation:

(15) $$M_{cell}(t)=M_{cell0}\cdot f(t)\cdot \exp(-\alpha (E^{external})(1-\delta (E^{external}-E_{\min}))t),$$

where δ is the delta function defined by the following conditions:

(16) $$\delta (E^{external}-E_{\min})=\left\{\begin{array}{l} 1,\;\;\;E^{external}=E_{\min}\;\\ 0,\;\;\;E^{external}>E_{\min}\;\;\mathrm{and}\;\;E^{external}<E_{\min} \end{array}\right.,$$

The process of cell proliferation is carried out in a thermostat, i.e., at a constant temperature. Thus, the process of cell proliferation can be considered isothermal. With a good approximation, it can be considered that the total mass of cell structure remains constant, since the growth of cells is accompanied by the consumption of nutrient medium.

It should be noted that, in thermodynamics, it is not the absolute values of thermodynamic parameters that are important, but their increments. Following this principle, we subtract Equation (11a) from Equation (11b) and, assuming as a hypothesis approximate equality of coefficients $a_{d}$ and $\;a_{d}^{*}$ at not too significant external energy influence, we obtain the following equation:

(17) $$\Delta E^{ext}=a_{d}({\tilde{D}}_{control}{\left(1/\rho_{\min}-1/\rho_{\max}\right)}_{control}-{\tilde{D}}_{ext}(1/\rho_{\min}-1/\rho_{\max})),$$

By using the experimental values of the electromagnetic pulse energy and changing values of Hausdorff’s dimension of culture, the averaged value $a_{d}$ at a fixed value of proliferation time and constant temperature (*T*) is determined. However, on the other hand, according to the second law of thermodynamics, in the difference form, taking into account the introduced measure of cell proliferation, we have:

(18) $$\Delta H\geq \frac{\Delta E^{external}}{a_{h}T},$$

where $a_{h}$ is the dimensional coefficient. The equivalent of combining the first and second laws of thermodynamics for the organized cellular structure will be the following inequality:

(19) $$\Delta H\geq \beta_{d}{}_{h}({\tilde{D}}_{control}{\left(1/\rho_{\min}-1/\rho_{\max}\right)}_{control}-{\tilde{D}}_{ext}(1/\rho_{\min}-1/\rho_{\max})).$$

This inequality (19) connects the entropy of cell proliferation, the surface density of cells, and the structural criterion of culture—Hausdorff’s dimension ($\beta_{dh}=a_{d}/a_{h})$.

The greater sign in (19) corresponds to an irreversible process, and the equal sign corresponds to a reversible process. It should be noted that the thermodynamic method allows us to transform a non-equilibrium process into a series of quasi-equilibrium processes if the initial and final states coincide.

## 3. Results

Table 1 shows the results of fibroblast’s proliferation: cell number growth, proliferation index *IP*, and doubling time under the longitudinal magnetic impulse action E= 500 J.

**Figure 1:**
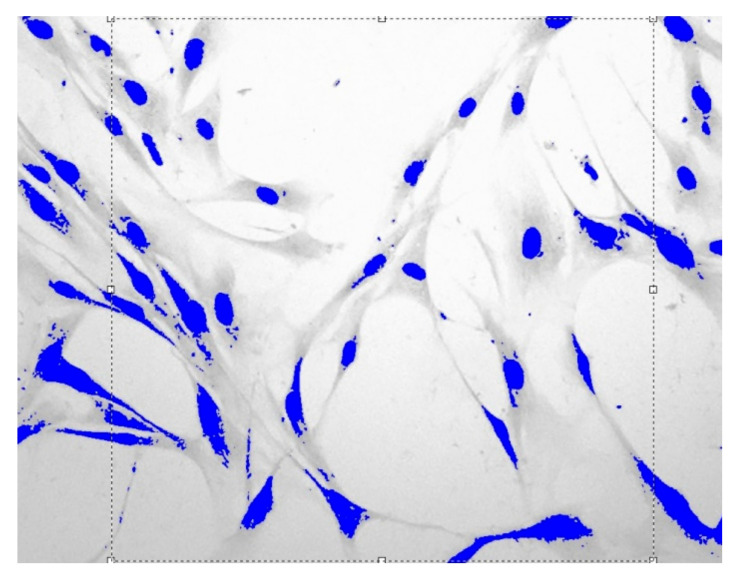
Fibroblasts E = 500 J, t = one day, D = 1.559 ± 0.0061, Sudan IV and hematoxylin-eosin staining, magnification n = 200.

**Figure 2:**
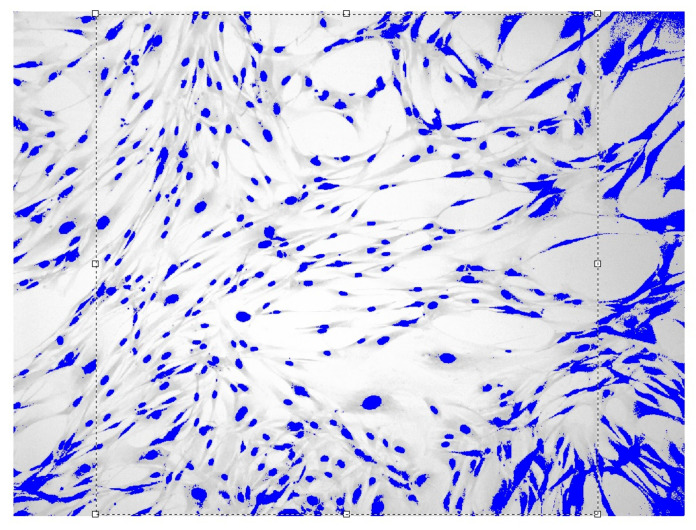
Fibroblasts E = 500 J, t = three days, D = 1.571 ± 0.034 Sudan IV and hematoxylin-eosin staining, magnification n = 200.

**Figure 3:**
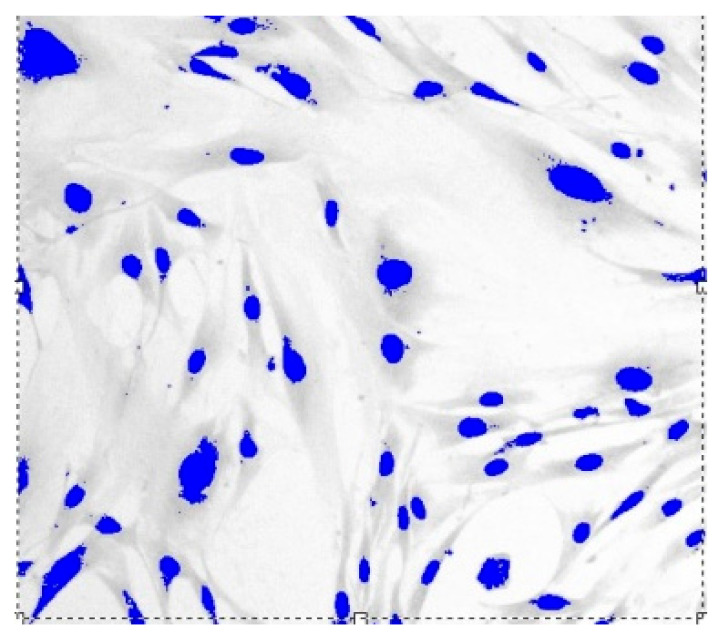
Fibroblasts E = 500 J, t = three days, D = 1.571 ± 0.0081, Sudan IV and hematoxylin-eosin staining, magnification n = 200.

**Figure 4:**
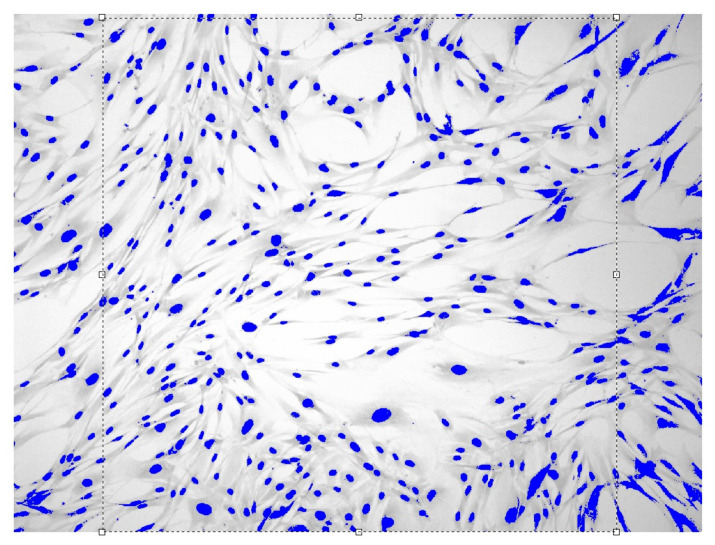
Fibroblasts E = 500 J, t = six days, D = 1.649 ± 0.0104, Sudan IV and hematoxylin-eosin staining, magnification n = 100.

Images of fibroblast culture at different time intervals of cell proliferation process at fixed various magnifications n are shown in Figure 1, Figure 2, Figure 3 and Figure 4.

The analysis of obtained results allows us to state that the organized cell structure of human dermal cells represents a fractal. The discrepancy in the values of Hausdorff’s criterion for the control culture and the culture subjected to pulsed magnetic field does not exceed 2.6% (Table 2).

The study of the control culture of fibroblasts indicates its homogeneity (t = 1 day D = 1.585 ± 0.054; t = 7 day = 1.86 ± 0.047 increase 100) (Table 3). The results obtained prove the possibility of using Hausdorff’s criterion for the analysis of proliferative activity of human dermal fibroblasts.

Based on the experimental data of fibroblast’s proliferation by using the algorithm of calculation, the cell proliferation entropy (6, 7), and fractal dimension of the culture (9), we have obtained the corresponding criterion assessments (Table 4).

As it follows from Table 4 with pulsed magnetic exposure E = 100 J, on average, fibroblast proliferation is higher than in control culture. Increase in pulse irradiation up to E = 500 J leads to significant death of fibroblasts (almost twice as much as in the control culture), and further increase in pulse irradiation up to E = 1000 J states complete destruction of cells in the irradiation zone (irradiation zone $d=10^{-2}$ m at the diameter of the alveolus $d=3\times 10^{-2}$ m). It is important to note that no differences in fibroblast density were observed in pulsed irradiation outside the irradiation zone $d>10^{-2}$ m as compared to the control culture.

**Figure 5:**
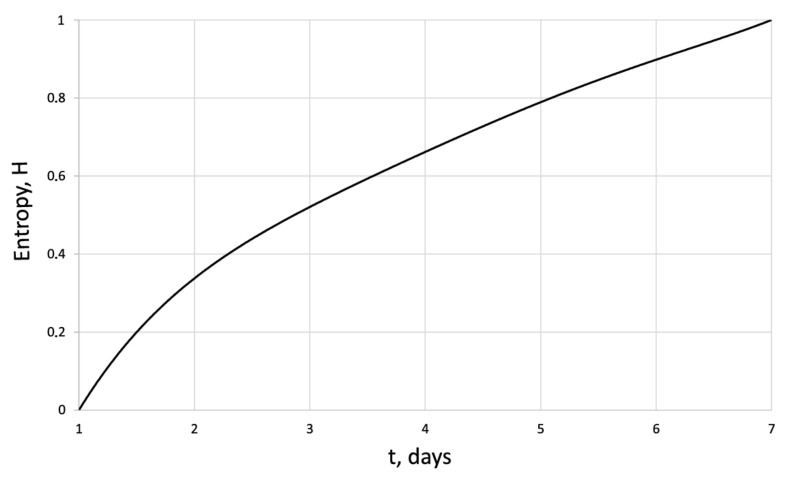
Entropy of the human dermal fibroblasts in control series.

**Figure 6:**
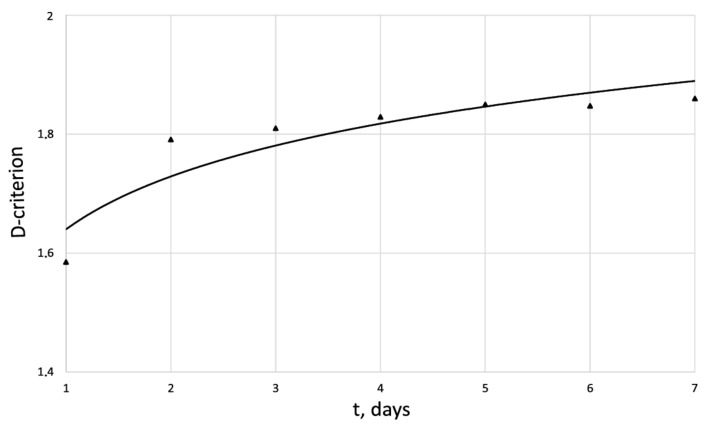
Dynamics of D-criterion of the human dermal fibroblasts in the control series.

**Figure 7:**
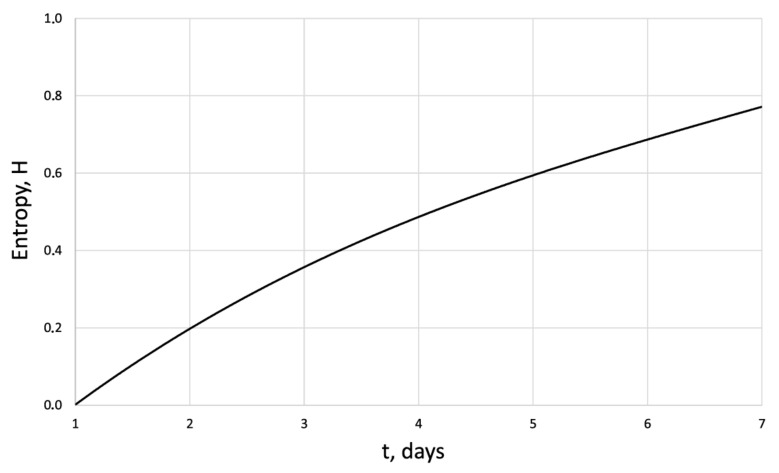
Entropy of the human dermal fibroblasts under pulsed electromagnetic influence E = 500 J.

**Figure 8:**
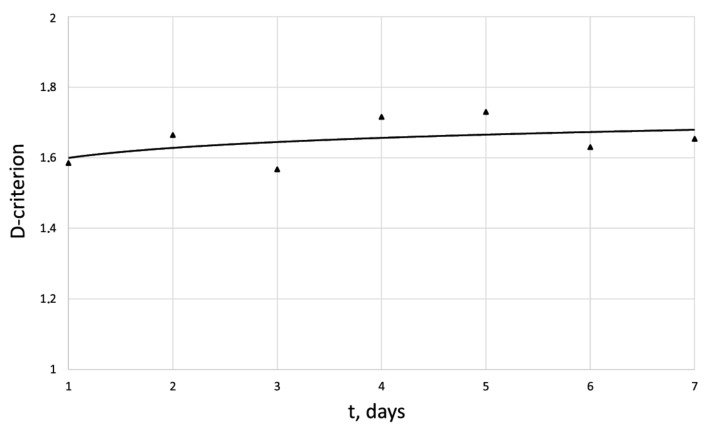
Dynamics of D-criterion for the human dermal fibroblast under pulsed electromagnetic exposure E = 500 J.

Figure 5, Figure 6, Figure 7 and Figure 8 show the corresponding graphical representations of the dynamics of entropy and fractal dimension of fibroblast culture at different values of electromagnetic pulse exposure.

The entropy trend of fibroblast proliferation makes it possible to give a criterial assessment of the process stability based on I. Prigogine’s theorem on entropy production minimum [26]: convex entropy trend—stable process, concave—unstable, linear trend—neutral stability. At neutral stability, any external perturbation can move the process to an unstable mode.

## 4. Discussion

An innovative method for studying the proliferation of cell structures based on the information–thermodynamic approach has been proposed. The developed information–thermodynamic method includes an algorithm for constructing deterministic entropies [27] and determining the criterion of cell structure—the criterion of fractality—Hausdorff’s dimension [29,30,31]. The first criterion, entropy, makes it possible to assess stability based on I. Prigogine’s theorem on the minimum entropy production [26] of the cell proliferation process under various influences, and the second criterion, Hausdorff’s dimension, determines the efficiency of cell culture transformation, including cells and intercellular space. In this case, these criteria (entropy and the Hausdorff’s criterion) simultaneously play the role of static and dynamic criteria for the proliferative activity of an organized cellular environment. Thus, static estimates of the state of the cell structure based on the entropy criteria and the Hausdorff’s criterion determine the deviation of these criteria from their reference values. The dynamic assessments of cell proliferation based on, for example, entropy, are connected by I. Prigogine’s theorem on the minimum entropy production [26]—the form of curvature of the entropy trend determines the stability of cell proliferation (entropy trend is convex—the process of proliferation of dermal fibroblasts is stable—and, if the trend is concave—the process is unstable).

The developed information–thermodynamic criteria represent, in a certain sense, the necessary and sufficient conditions for evaluating the efficiency of cell culture proliferation in the absence and presence of external influence (drug, electromagnetic, etc.) on the culture. An energy balance equation for an organized cellular structure is derived, which makes it possible to find a thermodynamic relationship between the mathematical measure of cell proliferation (H) and the criterion of an organized cellular structure (D). For the first time, on the basis of the energy balance equation, an analogue of the second law of thermodynamics for an organized cellular structure was formulated, which determines the relationship in the form of an inequality between the entropy increment (H) and the Hausdorff’s dimension criterion (D). This result testifies to the fact that the Hausdorff’s criterion (D) is a thermodynamic criterion that correlates with the energy supplied to the cell structure. This fact gives grounds for the development of a single energy equivalent of the impact (electromagnetic, chemotherapeutic, etc.) on the proliferative activity of dermal cells. In this case, these criteria (entropy and the Hausdorff’s criterion) simultaneously play the role of static and dynamic criteria for the proliferative activity of an organized cellular environment. Thus, static estimates of the state of the cell structure based on the entropy criteria and Hausdorff’s criterion determine the deviation of these criteria from their reference values. The dynamic assessment of cell proliferation based, for example, on entropy is connected by I. Prigogine’s theorem on the minimum entropy production [26]—the form of curvature of the entropy trend determines the stability of cell proliferation. The developed information-thermodynamic criteria represent, in a certain sense, the necessary and sufficient conditions for evaluating the efficiency of cell culture proliferation in the absence and presence of external influence (drug, electromagnetic, etc.) on the culture.

This fact gives grounds for the development of a single energy equivalent of the impact (electromagnetic, chemotherapeutic, etc.) on the proliferative activity of dermal cells. The developed information–thermodynamic method was tested in the cultivation of juvenile dermal fibroblasts under electromagnetic exposure. An analysis of the results obtained allows us to state that the organized cellular structure of human dermis cells is a fractal. This experimental fact makes it possible to use the tools of synergetics and fractal theory for advanced studies of organized cellular structures. We would prefer to suggest that the developed entropy–structural (entropy–fractal) apparatus (3–19) can become an innovative toolkit for further development of three-dimensional bioprinting technologies with specified structural conditions [38,39,40,41,42,43,44]. It should be noted that the developed method is promising for the analysis of the proliferative activity of cancer cells under various influences. This method, based on the analytical apparatus of phenomenological and non-equilibrium thermodynamics, creates the basis for the development of technology for precision (personalized) exposure (electromagnetic, chemotherapeutic, radiation, etc.) in oncological diseases.

## Figures and Tables

**Table 1:** Proliferative activity of the culture of dermal fibroblasts (electromagnetic impulse action E=  500 J).

Term of Experiment, Sowing Day/Experiment Day after Exposure	Monolayer Density, Cell/1 mm^2^	IP, Rel. Units	Time Doubling, h	Quantity Doublings, Rel. Units
Initial data (1 day after sowing)	42.9 ± 5.2			
2 days/1 day	114.9 ± 7.5	2.6	17.4	
3 days/2 days	134.7 ± 6.4	1.1	106.3	
4 days/3 days	163.3 ± 17.5	1.3	93	
5 days/4 days	208.9 ± 18.1	1.1	69.5	
6 days/5 days	263.3 ± 15.6	1.1	74.4	
7 days/6 days	338.6 ± 25.3	1.2	68.3	3

**Table 2:** Criterial assessment of the cell structure fractality.

t, Days	D-Criterion	E, J	Increasing n
1	1.55	500	100
1	1.58	500	200
1	1.51	500	400
6	1.65	500	100
6	1.59	500	200
6	1.67	500	400

$D_{everage}$ = 1.546 ± 0.036 t = 1 day ε=2.6%; $D_{everage}$ = 1.633 ± 0.043 t = 6 days ε=2.6%.

**Table 3:** Criterion assessment of the cell structure homogeneity.

t, Day	D-Criterion	E, J	Increasing n
1	1.56	500	200
1	1.55	500	200
1	1.60	500	200
1	1.55	500	200
1	1.52	500	200
1	1.55	500	200
1	1.56	500	200

$D_{everage}$ = 1.559 ± 0.041 t = 1 day ε=2.6%.

**Table 4:** Dynamics of entropy–structural criteria during pulsed magnetic action on human dermal fibroblasts.

E = 500 J							
Days	1	2	3	4	5	6	7
ρ	42.8	114	134.7	163.2	208	263	338.5
D	1.58	1.56	1.57	1.71	1.72	1.62	1.67
H	0	0.41	0.48	0.54	0.64	0.72	0.8
E = 500 J							
Days	1	2	3	4	5	6	7
ρ	42.2	132.1	201.33	385.2	426.7	499.7	562.8
D	1.58	1.80	1.69	1.87	1.79	1.81	1.86
H	0	0.48	0.62	0.84	0.87	0.92	0.96
Control							
Days	1	2	3	4	5	6	7
ρ	44	90.7	142	201	286.5	442.7	630.5
D	1.58	1.79	1.81	1.82	1.85	1.84	1.86
H	0	0.34	0.50	0.57	0.7	0.86	1

## Data Availability

The authors confirm that the data supporting the findings of this study are available within the article. Raw data that support the findings of this study are available from the corresponding author, upon reasonable request.

## Abbreviations

List of abbreviations: CMF, VMF, and PMF—constant, variable, and pulsed magnetic field, respectively.

## References

[1] Gluschenkov V.A., Volova L.T., Belyaeva I.A., Boltovskaya V.V., Rossinskaya V.V., Ignatenko A.I., Nefedova I.F., Kulagina L.N. (2020). Impact of pulsed magnetic field of high intensity on human dermal fibroblasts in culture. Tidings Samara Sci. Cent. Russ. Acad. Sci..

[2] Rybakov U.L., Kizhaev E.V., Letyagin V.P., Nikolaeva T.G. (2005). System-wide Magnetotherapy in Oncology. Med. Phys..

[3] Rudykina O.A., Grekhov R.A., Suleimanova G.P., Adamovich E.I. (2016). Electromagnetic field and its influence on physiological processes in human body. Vest. Volgogr. State Univ. Ser. 11 Nat. Sci..

[4] Perov S.Y. (2015). The study of the functional state of individual systems of the body under the influence of low-intensity radiofrequency electromagnetic field. Bull. New Med. Technol..

[5] Pletnev A.S. (2016). Magnetic Fields in Experimental and Clinical Oncology.

[6] France E.M. (2019). Antitumor effect of magnetic fields and their effect on pain in experimental and clinical oncology. Res. Pract. Med..

[7] Maximov A.V., Kiryanova V.V., Maximova M.A. (2013). Therapeutic use of magnetic fields. Physiother. Balneol. Rehabil..

[8] Gluschenkov V.A. (2014). Technology of Magnetic Pulse Processing of Materials.

[9] Rubner M. (1908). Das Problem der Lebensdauer und Seine Beziehungen zu Wachstum und Ernährung.

[10] Bauer E.S. (1935). Theoretical Biology.

[11] Backman G. (1943). Wachstum und Organiche Zeit.

[12] Du Noüy L.P. (1936). Biological Time.

[13] Brody D.A., Cox J.W., Horan L.G. (1973). Hexadecapolar shift equations applicable to equivalent cardiac generators of lower degree. Ann. Biomed. Eng..

[14] Brody S. (1945). Bioenergetics and Growth.

[15] Zotin A.I. (1974). Thermodynamic Approach to the Problems of Mothers of Development, Growth and Aging.

[16] Zotin A.I. (1982). Biology of Aging.

[17] Zotin A.I. (1988). Thermodynamic Basis of the Influence of Organs on External and Internal Factors.

[18] Zotin A.I., Zotin A.A. (1996). The Thermodynamic Foundation of Development. Ontogenez.

[19] Zotin A.I., Zotina R.S. (1993). Phenomenological Theory of Development, Growth, and Aging of the Organism.

[20] Zotin A.I., Alekseeva T.A. (1984). Rubner’s constant as a criterion for species lifespan. Fiziol. Mag..

[21] Zotin A.A. (2001). Vladimirova IG. Respiration rate and species-specific lifespan in fresh water bivalves of Margaritiferidae and Unionidae families. Izv. Akad. Nauk Ser. Biol..

[22] Alimov A.F., Kazantseva T.I. (2007). Definition of unit internal (physiological) time. Biol. Bull..

[23] Alimov A.F., Kazantseva T.I. (2004). The basic life history parameters and their relationships. Zhurnal Obs. Biol..

[24] Reiss J.O. (1989). The meaning of developmental time: A metric for comparative embryology. Am. Nat..

[25] Boddington M.J. (1978). An absolute metabolic scope for activity. J. Theor. Biol..

[26] Prigogine I., Glansdorff P. (1973). Thermodynamic Theory of Structure, Stability and Fluctuations.

[27] Volov V.T. (2021). Hypothesis of the Entropy Invariant for Biological Organisms. Biophysics.

[28] Martin N.F., England J.W. (2011). Mathematical Theory of Entropy.

[29] Feder J. (2013). Fractals.

[30] Terekhov S.V. (2011). Fractals and Physics of Similarity.

[31] Mandelbrot B.B., Mandelbrot B.B. (1982). The Fractal Geometry of Nature.

[32] Greenberg K.N. (1988). Cultivation of human fibroblasts for diagnostics of hereditary diseases. Methods of Cell Cultivation: Collection of Scientific Works.

[33] Volova L.T., Pugachev E.I., Rossinskaya V.V., Boltovskaya V.V., Dolgushkin D.A., Ossina N. (2020). Rheumatoid Arthritis: Applicability of Ready-to-Use Human Cartilaginous Cells for Screening of Compounds with TNF-Alpha Inhibitory Activity. Biomolecules.

[34] Osina N.K., Pugachev E.I., Koliadenko I.A., Pryazhkina V.V., Shakurov E.G., Orlov E.V., Volova L.T. (2021). In vitro test system for screening drugs with IL-17A inhibitory activity. Genes Cells.

[35] Zorin V., Zorina A., Petrakova O., Cherkasov V. (2009). Dermal Fibroblasts for Treatment of Skin Defects. Genes Cells.

[36] Kuzmicheva V.I., Volova L.T., Gilmiyarova F.N., Bykov I.M., Avdeeva E.V., Kolotieva N.A. (2020). Fibroblasts as an object for studying proliferative activity in vitro. Sci. Innov. Med..

[37] Kotelnikov G.P., Kolsanov A.V., Nikolaenko A.N., Volova L.T., Rossinskaya V.V., Boltovskaya V.V., Popov N.V., Shcherbovskikh A.E., Prikhodko S. (2018). Testing of additive materials on human fibroblast cell cultures. Clin. Exp. Surg. J. Acad. B.V. Petrovsk..

[38] Coburn J. (2017). FDA Additive Manufacturing Working Group. Technical Considerations for Additive Manufactured Devices. FDA/RSNA Joint Meeting on 3D Printed Patient-Specific Anatomic Models.

[39] Hesuani Y.D., Pereira F.D.A.S., Parfenov V., Koudan E., Mitryashkin A., Replyanski N., Kasyanov V., Knyazeva A., Bulanova E., Mironov V. (2016). Design and implementation of novel multifunctional 3D bioprinter. 3D Print. Addit. Manuf..

[40] Shafiee A., Atala A. (2016). Printing Technologies for Medical Applications. Trends Mol. Med..

[41] Leberfinger A.N., Ravnic D.J., Dhawan A., Ozbolat I.T. (2017). Concise Review: Bioprinting of Stem Cells for Transplantable Tissue Fabrication. Stem Cells Transl. Med..

[42] Rocca M., Fragasso A., Liu W., Heinrich M.A., Zhang Y.S. (2017). Embedded Multimaterial Extrusion Bioprinting. SLAS Technol..

[43] Mandrycky C., Wang Z., Kim K., Kim D.H. (2016). 3D bioprinting for engineering complex tissues. Biotechnol. Adv..

[44] Boyd-Moss M., Fox K., Brandt M., Nisbet D., Williams R. (2017). Bioprinting and Biofabrication with Peptide and Protein Biomaterials. Adv. Exp. Med. Biol..

